# Adult Attachment Questionnaire: evidence of validity in a sample of IPVAW offenders

**DOI:** 10.3389/fpsyg.2024.1265303

**Published:** 2024-02-02

**Authors:** Iria de la Osa-Subtil, Andrés Arias Astray, Pedro Vicente Mateo Fernandez, María José de Dios-Duarte

**Affiliations:** ^1^Faculty of Psychology, Complutense University of Madrid, Madrid, Spain; ^2^Social Work Department, Knowledge Technology Institute, Faculty of Social Work, Complutense University of Madrid, Madrid, Spain; ^3^Nursing Department, Faculty of Nursing, University of Valladolid, Valladolid, Spain

**Keywords:** IPVAW, attachment, evidence of validity, psychometric properties, questionnaire

## Abstract

**Introduction:**

Adult attachment can be understood as a cognitive and emotional system concerning oneself and others, based on previous attachment experiences throughout life. This system automatically affects relationships with others. Because of its importance in the interpersonal domain, it has been studied on numerous occasions in research on intimate partner violence. The aim of this study was to obtain evidence of validity of the Adult Attachment Questionnaire (AAQ) in a sample of 331 men convicted of intimate partner violence against women (IPVAW).

**Methods:**

The AAQ assesses adult attachment style in four dimensions that, together, yield four attachment categories. A psychometric analysis was performed, including reliability analysis and confirmatory factor analysis of the items, which ratified the factorial structure of the questionnaire. For a correct fit of the model, it was necessary to eliminate 4 of the items from the original scale.

**Results:**

A latent profile analysis was also carried out, which identified four attachment styles: secure, preoccupied/anxious, avoidant/dismissing and fearful. Reliability indices were adequate. In general, the attachment profiles obtained ranges and means similar to those found in the general population study. The distribution of attachment styles was not equal: 50.57% of the participants presented secure attachment, 4.57% avoidant/dismissing attachment, 36.9% preoccupied/anxious, and 8.57% fearful.

**Discussion:**

In conclusion, a valid and reliable instrument was determined based on the original AAQ scale to measure attachment in men convicted of IPVAW.

## 1 Introduction

Adult attachment refers to the affective bond that is established between adults in the context of intimate relationships. It is an automatic response based on cognitive-emotional schemas that develop by virtue of attachment experiences with significant figures throughout life (Feeney and Noller, 1996; Feeney, 2002).

Adult attachment can be categorically classified by the combination of two dimensions with negative and positive poles: cognitive schemas about oneself (resulting in a spectrum between high and low relationship anxiety) and cognitive schemas about others (resulting in a spectrum between high and low relationship avoidance). The secure style is identified by having a positive mental model and confidence in oneself and others, high self-esteem, no serious interpersonal problems, and a desire for intimacy. Intimacy, that is, in which the person feels comfortable (Bartholomew and Horowitz, 1991; Mikulincer and Horesh, 1999). In addition, these are individuals who maintain a balance between their affective needs and personal autonomy (Mayseless, 1996), which usually leads to fulfilling personal relationships. In contrast, in the fearful style a negative mental model of both self and others and low confidence in both cases prevails. These are individuals who frequently need the approval of other people, consider relationships as secondary to professional matters, and feel uncomfortable in intimate contexts (Bartholomew and Horowitz, 1991; Mayseless, 1996). In the avoidant/dismissing style the prevailing mental model of self is positive, but negative toward others (Bartholomew and Horowitz, 1991). This style is characterized by high emotional self-sufficiency and low activation of attachment needs. These individuals consider personal interactions as secondary to material matters and are very uncomfortable in intimate circumstances (Mayseless, 1996). Finally, people with a preoccupied/anxious affective style present a negative mental model of themselves but a positive model of others (Bartholomew and Horowitz, 1991). Their self-esteem is low, they present high activation of the attachment system and dependency behaviors. These subjects show a constant need for approval and an excessive preoccupation with social interactions.

The categorical paradigm for understanding attachment theory has disadvantages compared to the dimensional one (Fearon and Roisman, 2017). Categorical measurement assumes that there are a limited number of attachment types and that these fit into a taxonomic system. It appears that dimensional measures fit the data better and have more predictive power over other variables. However, this drawback can be addressed by understanding that categorical and dimensional measures capture different aspects of attachment: the overall strategy and the relative strength of attachment behavioral systems, with the categorical perspective being an indicator of the predominant strategy of the subject (Cowan and Cowan, 2007).

Several scales have been developed to assess attachment style in the general population. Among the best known are the Relationship Questionnaire (RQ; Bartholomew and Horowitz, 1991) and the Experiences in Close Relationships Scale (ECR; Brennan et al., 1998 adapted to Spanish by Alonso-arbiol et al., 2007). The RQ consists of choosing one of the four paragraphs that represent the four attachment styles: secure, fearful, preoccupied/anxious and avoidant/dismissing. It is a 36-item questionnaire (where responses range from 1: strongly disagree to 7: strongly agree) that measures two dimensions of insecurity: avoidance and anxiety. Based on these scales, a questionnaire was developed to assess attachment style in the Spanish context: the Adult Attachment Questionnaire (AAQ; Cuestionario de Apego Adulto in Spanish). This questionnaire was created by Melero and Cantero (2008), in order to supplement the Relationship Questionnaire with a dimensional measure of attachment. This measure is based on theoretical constructs derived from qualitative research on the characteristics of the different styles. The AAQ consists of 40 items that are grouped into four subscales, measuring aspects such as self-concept, trust in others, need for approval, independence/autonomy/self-sufficiency, viewing relationships as secondary concerns, expression of feelings, discomfort with intimacy, conflict resolution strategies, dissatisfaction in relationships, achievement orientation vs. personal orientation, fear of relationships, and interpersonal problems. From the scores obtained in the four subscales, the prevailing attachment style in the subject is estimated, according to the Relationship Questionnaire.

Given the relational nature of attachment style, it impacts the romantic sphere as it determines our expectations, needs and behaviors in love (Simpson et al., 2007). It has also been related to relationship satisfaction, health, and wellbeing (Leak and Cooney, 2001). In addition, the way we bond influences communication patterns and the adaptive or maladaptive conflict resolution strategies used (Mikulincer and Shaver, 2011; Paquette et al., 2020). Consequently, it is of great interest to know attachment style when analyzing interpersonal violence. Attachment style gives rise to functional or dysfunctional expression of anger, domestic and intimate partner violence, criminal and antisocial behavior, and even intergroup violence (Mikulincer and Shaver, 2011).

Thus, partner violence, explained from the attachment point of view, is understood as an exaggerated response to the perception of a partner's hurtful behavior (Mikulincer and Shaver, 2007). It involves the concentration of behaviors aimed at avoiding relationship breakdown (Dutton et al., 1994; Bartholomew and Allison, 2006). Thus, the affective style of secure attachment is related to a greater use of prosocial skills (Mikulincer and Shaver, 2011). In contrast, people with insecure attachment tend to have problems in their intimate relationships, such as being sad, angry, jealous, and hostile toward their partners (Dutton et al., 1994). Affective attachment style is a risk variable that, in interaction with others, predicts different forms and degrees of intensity of intimate partner violence against women (IPVAW) (de la Osa et al., 2022).

Given that validity evidence is limited to scores obtained for a specific use and under specific conditions (Messick, 1993), it cannot be assumed that an instrument that has been validated in a general or clinical population is equally valid for specific populations such as convicted men, which presents very specific characteristics and needs. It is essential to be aware of the psychometric properties of instruments that are used in investigations or evaluations related to the legal context (Kennedy et al., 2019).

### 1.1 Aims

The aim of this study was to identify different evidence of validity of the AAQ in men convicted of IPVAW in Spain. To this end, the following specific objectives were proposed: (a) to determine the psychometric properties of the items that make up the questionnaire, (b) to check whether the structure of the questionnaire for the general population coincides with the structure for measuring the construct of abusers, (c) to determine the reliability of its four subscales, and (d) to confirm whether the combination of the subscales generates groupings theoretically compatible with attachment styles.

## 2 Methods

### 2.1 Participants and procedure

This study involved 331 men, convicted of an IPVAW crime in Spain, who were serving an alternate or suspended sentence or were incarcerated. The age range of the participants was between 19 and 72 years (M = 39.8, SD = 11.2). The 76.6% of participants were European, with 70% of the total sample being Spanish, 18% Latin American, 4.3% African, and 1.1% Asian. Some 28.4% of the participants had primary education, 53.0% had secondary education, 12.5% had university studies, and 6.1% had no studies. Twenty-two percentage of the participants considered themselves to be of low socioeconomic class, 59.2% middle class, 10.9% upper middle class, and 5.5% high class.

The exclusion criteria established in this study were: having served the sentence, not having been previously convicted, being a minor, and not knowing how to read or not understanding Spanish correctly. It was no necessary to exclude any participant.

The sampling procedure used to select participants was based on a non-probabilistic convenience approach. The design of this study was observational, analytical, prospective and cross-sectional. The evaluation protocol was implemented during the presentation sessions of an intervention programme aimed at men convicted of gender violence, pursuant to Organic Law 1/2004 on Comprehensive Protection Measures against Gender Violence. Before completing the questionnaires, the participants received information about the study verbally, as well as an information sheet and an informed consent form that they had to sign in order to participate. Participation in this study was voluntary and disinterested.

This study obtained a favorable report from the ethics committee of the Faculty of Psychology of the Complutense University and authorization from the General Secretariat of Penitentiary Institutions of Spain.

### 2.2 Instruments

#### 2.2.1 Sociodemographic questionnaire

A questionnaire was created *ad-hoc* to assess the sociodemographic and personal characteristics of the participants, including age, nationality, and level of education.

#### 2.2.2 Adult Attachment Questionnaire (AAQ)

The Adult Attachment Questionnaire (AAQ; Melero and Cantero, 2008) consists of 40 Likert-type items (1–6) that assess different dimensions of attachment in adults. These items are part of a latent structure of four factors that, grouped together, give rise to the theorized attachment styles, both bidimensional (secure and insecure) and categorical (secure, preoccupied, fearful, avoidant). The subscales are: subscale 1: low self-esteem, need for approval and fear of rejection; subscale 2: hostile conflict resolution, resentment and possessiveness; subscale 3: expression of feelings and comfort with relationships; subscale 4: emotional self-sufficiency and discomfort with intimacy.

The questionnaire presented adequate internal consistency in the first three subscales (α_scale1_ = 0.86, α_scale2_ = 0.80, α_scale3_ = 0.77), but not in the fourth (α_scale4_ = 0.68).

Content validity was ensured through the selection and review of items by experts in the field of attachment, guaranteeing that the questionnaire accurately reflected the theoretical dimensions of adult attachment. To gather evidence about the construct, an exploratory factor analysis (orthogonal rotation), a reliability analysis with Cronbach's α test and a K-Means cluster analysis were performed. These analyses have shown that the questionnaire items are grouped according to the theoretical dimensions of adult attachment, thus confirming the construct validity of the instrument.

### 2.3 Analysis

RStudio 4.2.3 was used to analyse the data. Descriptive analyses were performed to characterize the sample. A descriptive analysis of the items was performed and their discrimination index was calculated. The internal consistency of each of the AAQ's four subscales was evaluated using the Omega McDonald coefficient (ω) and confidence intervals (Viladrich et al., 2017). Acceptable (>0.70), good (>0.80), and excellent (>0.90) results were obtained (Taber, 2018). The four-factor model was then tested using Confirmatory Factor Analysis (CFA). The Unweighted Least Squares (ULS) estimator was chosen. This parameter estimator is recommended for categorical variables, does not require a specific distribution, is suitable for small samples, *n* = 200 (Muthén, 1983; Batista-Foguet and Coenders, 2000; Brown, 2015), and obtains better results with ordinal data than the Maximum Likelihood Estimator (Li, 2016) and Diagonally Weighted Least Square (Forero et al., 2009). The analyses were based on the polychoric correlation matrix. Items that presented factor saturations lower than 0.4 were eliminated (Byrne, 2013). The scale was evaluated using the goodness of fit indices (Hooper et al., 2008): Comparative Fix Index >0.90 (Bentler, 1990) and Tucker Lewis Index >0.90 (TLI, Tucker and Lewis, 1973), Root Mean Square Error of Approximation ≤ 0.08 (RMSEA, Steiger, 1990): Good fit ≤ 0.05, acceptable fit between 0.05 and 0.08; and Standarized Root Mean Residual (SRMR, Fan and Sivo, 2007): Good fit ≤ 0.05, acceptable fit between 0.05 and 0.08.

A latent profile analysis (LPA) with the packages “mclust” and “lpa” was used to determine the number of existing groups according to the four subscales of the AAQ. Class selection criteria were based on model fit. The fit was assessed by reviewing the variations in entropy, considering that the lower this data, the less clear the separation between groups, the minimum acceptable being 0.70 (Lanza and Cooper, 2016). The decline in the Log Likelihood Logarithm (LogLik), the Akaike Information Criterion (AIC; Akaike, 1987), the Bayesian Information Criterion (BIC; Schwarz, 1978) and the BIC adjusted to the sample size were also assessed, the last three being acceptable from a value of 0.90. Subsequently, the interpretability criterion was taken into account, given that the profile solution must make theoretical sense to be useful (Muthén and Muthén, 2000). Finally, an analysis of variance was performed to determine the differences between the profiles obtained and the four subscales of the AAQ and thus determine which of the profiles corresponds to the attachment styles.

### 2.4 Results

#### 2.4.1 Confirmatory factor analysis

**Figure 1:**
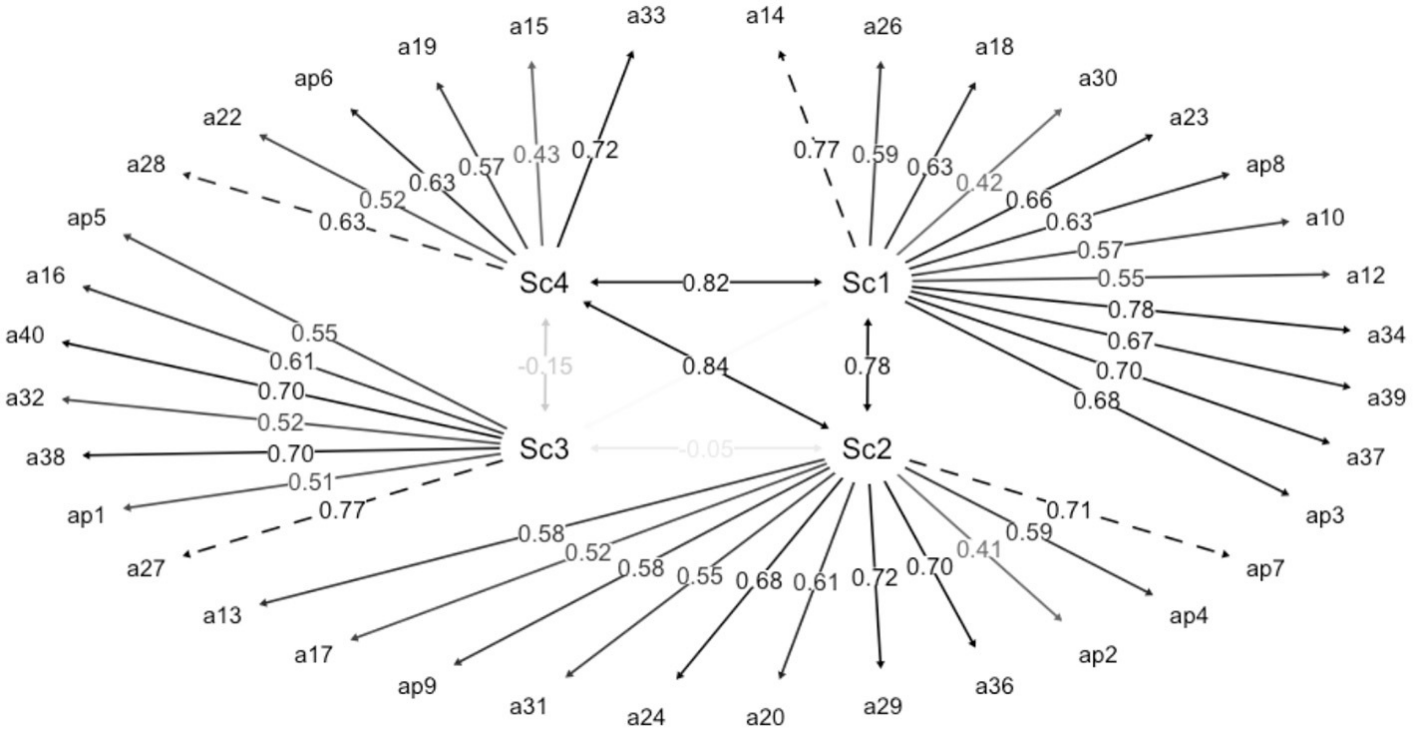
Path diagram of the model with the standardized weights of the model 2.

First, a model was estimated using all of the items (see Table 1) and with the structure proposed by Melero and Cantero (2008). A total of 269 parameters were estimated with 736 degrees of freedom, data that indicate that it is correctly identified. The metric for items 7, 14, 27, and 28 was set to 1 since they were the most saturated and were statistically significant (see Table 2). The model results showed a moderately adequate (unsatisfactory) 4-factor model fit [CFI = 0.887; TLI = 0.880; RMSEA = 0.093 [0.090; 0.097]; and SRMR = 0.101]. The study of factor saturations showed four items that saturated below 0.40. Item 21 (“*I am self-confident”)* of subscale 1, items 11 (“*I have trouble asking personal questions”)* and 35 (“*I am a person who prefers solitude to social relationships*”) of subscale 3, and item 25 of subscale 4 (“*I prefer stable relationships to sporadic partners”*). Once these four items were removed, a properly identified model (see Figure 1) with 258 parameters and 659 degrees of freedom was obtained. The fit indices improved, thus becoming acceptable [CFI = 0.942; TLI = 0.938; RMSEA = 0.073 [0.069; 0.077]; and SRMR = 0.085].

**Table 1:** Psychometric properties of the items.

**Item**	**M**	**SD**	**DI**	**Sk**	**Ku**
**Scale 1: low self-esteem, need for approval and fear of rejection**					
3	2.37	1.69	0.550	0.9106	−0.5389
8	1.96	1.51	0.414	1.5117	1.1081
10	2.17	1.59	0.473	1.1354	0.0460
12	3.05	1.77	0.508	0.2676	−1.2647
14	1.82	1.37	0.619	1.6511	1.6763
18	2.44	1.57	0.579	0.7166	−0.6941
21	2.45	1.74	0.064	0.9699	−0.4661
23	2.04	1.43	0.485	1.3052	0.6578
26	2.33	1.59	0.547	0.9426	−0.3087
30	3.67	1.78	0.394	−0.1682	−1.2436
34	1.85	1.42	0.523	1.6672	1.7139
37	2.29	1.64	0.521	0.9583	−0.4251
39	2.53	1.65	0.526	0.7041	−0.7696
**Scale 2: hostile conflict resolution, resentment, and possessiveness**					
2	2.57	1.57	0.377	0.7120	−0.5610
4	1.92	1.45	0.508	1.4900	1.0962
7	1.80	1.16	0.594	1.5004	1.6823
9	2.57	1.65	0.395	0.6228	−0.8665
13	2.00	1.58	0.386	1.4750	0.8794
17	2.46	1.52	0.434	0.6970	−0.6121
20	2.36	1.45	0.411	0.8233	−0.2475
24	1.89	1.36	0.567	1.6003	1.6831
29	1.82	1.21	0.521	1.5547	1.8296
31	2.25	1.57	0.441	1.0584	−0.0953
36	1.41	1.02	0.429	9.3376	3.0373
**Scale 3: expression of feelings and comfort with relationships**					
1	4.44	1.55	0.342	−0.8381	−0.2811
5	4.06	1.71	0.421	−0.5852	−0.8200
11	4.45	1.81	0.234	−0.7670	−0.8597
16	4.31	1.69	0.489	−0.7895	−0.5785
27	4.86	1.44	0.550	−1.3364	1.0323
32	4.69	1.57	0.422	−1.1031	0.1242
35	4.35	1.62	0.218	−0.6824	−0.9761
38	4.36	1.62	0.518	−0.7379	−0.5311
40	4.49	1.51	0.544	−1.0004	0.1961
**Scale 4: emotional self-sufficiency and discomfort with intimacy**					
6	1.86	1.43	0.374	1.6608	2.1937
15	3.22	1.81	0.254	0.0817	−1.3648
19	2.36	1.56	0.415	0.8308	−0.4840
22	1.82	1.27	0.413	1.8265	2.3200
25	2.71	1.93	0.107	0.6725	−1.1168
28	2.11	1.50	0.381	1.0936	−0.0349
33	1.73	1.35	0.396	1.8265	2.3200

M, mean; SD, standard deviation; DI, discrimination index based on item-test correlation; Sk, Skewness; Ku, Kurtosis.

**Table 2:** Comparison of model fit parameters with the different profiles.

**Number of profiles**	**LogLik**	**Entropy**	**BIC**	**SABIC**	**AIC**
1	−4,906	1.000	9,860	9,834	9,829
2	−4,763	0.803	9,602	9,561	9,552
3	−4,712	0.865	9,529	9,472	9,460
4	−4,673	0.819	9,482	9,409	9,393
5	−4,673	0.773	9,509	9,420	9,401
6	−4,671	0.667	9,535	9,430	9,408

LogLik, log likelihood; BIC, Bayesian information criterion; SABIC, sample-adjusted Bayesian information criterion; AIC, Akaike information criterion.

Regarding internal consistency, the ω values were 0.835 for subscale 1, 0.806 for subscale 2, 0.752 for subscale 3, and 0.641 for subscale 4. In the model with the 4 items eliminated, better reliability values were obtained (subscale 1 = 0.847, subscale 2 = 0.806, subscale 3 = 0.782, subscale 4 = 0.665), obtaining good values of internal consistency in subscales 1 and 2, and an acceptable value in subscale 3. The value of subscale 4 did not indicate acceptability.

#### 2.4.2 Latent profile analysis

Six latent profile analysis (LPA) models were estimated with solutions from 1 to 6 profiles. As found by the authors who constructed the original scale, both a 2-profile solution and a 4-profile solution were reasonable choices based on fit indices (see Table 2) and conceptual validity. Six latent profile analysis models were compared using entropy, BIC, sample-adjusted BIC (SABIC), AIC, and LogLik.

Specifically, the 4-profile model was chosen, which obtained generally low values in the fit indices (LogLik = −4,673 BIC = 9,482, SABIC = 9,409, AIC = 9,349) compared to the more and less class models. Furthermore, an acceptable entropy (0.819) was obtained, indicating a clear separation between the groups. Taking into account this decrease in the indices and the theoretical criteria, the membership of each subject to the four profiles was saved to later determine if they coincided with the attachment styles.

#### 2.4.3 Analysis of variance

**Figure 2:**
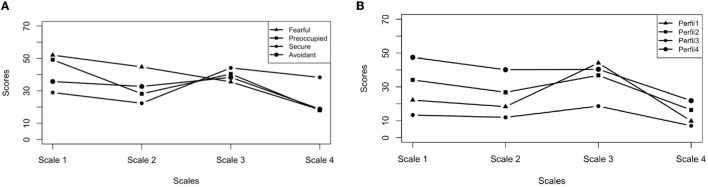
Means of the four attachment profiles and their scores in the four subscales obtained in the general population **(A)** by Melero and Cantero (2008) and those obtained in this research with batterers **(B)**.

An analysis of variance was performed to determine the mean scores of each profile in each of the subscales and compared with the results obtained by Melero and Cantero (2008). Subsequently, the attachment style corresponding to each profile was determined. The results showed significant differences in the four groups. The profile 1 (see Figure 2), was composed of 50.57% of the sample. This profile showed a secure attachment style, with higher mean scores on subscale 3 (M_Scale3_ = 44.15, SD_Scale3_ = 6.27, Range_Scale3_ = 9–54, *p* < 0.01) and lower mean scores on the other subscales (M_Scale1_ = 22.19, M_Scale2_ = 18.30, M_Scale3_ = 9.81), coinciding with the results of the original study (M = 40.07, Range = 14–54; M_Scale1_ = 28.94, M_Scale2_ = 22.34, M_Scale4_ = 13.99). Profile 2 comprised 36.29% of the sample. In this case the attachment style was preoccupied, with a high mean score in subscale 1 (M_Scale1_ = 34.14, SD_Scale1_ = 8.80, Range_Scale1_ = 12–43, *p* < 0.01) and moderate mean scores in the other subscales (M_Scale2_ = 26.79, M_Scale3_ = 36.86, M_Scale4_ = 16.33). These data were lower than those obtained in the general population (M_Scale1_ = 49.15, Range_Scale1_ = 15–77; M_Scale2_ = 28.17, M_Scale3_ = 40.40, M_Scale4_ = 17.97). Profile 4 consisted of 8.57% of the sample. Correspondence with fearful/hostile attachment was determined, with high mean scores on all subscales (M_Scale1_ = 47.47, SD_Scale1_ = 8.97, Range_Scale1_ = 26–53, *p* < 0.01; M_Scale2_ = 40.13, SD_Scale2_ = 8.72; M_Scale3_ = 40.40, SD_Scale3_ = 6.55; M_Scale4_ = 21.87, SD_Scale4_ = 4.73). Similarity to the original study was observed through the maximum variation of 5 points on subscale 3 (M_Scale1_ = 52, M_Scale2_ = 44.73, M_Scale3_ = 35.51, M_Scale4_ = 18.41). Finally, profile 3 appeared to be the avoidant attachment style, with 4.57% of the sample and a medium-high score on subscale 4 compared to the responses of the other participants (M_Scale4_ = 7.87, SD_Scale4_ = 4.73, Range_Scale4_ = 13–30, *p* < 0.01) and low/moderate mean scores on the other subscales (M_Scale1_ = 13.38, M_Scale2_ = 12.00, M_Scale3_ = 18.63). These results demonstrate a similar score on subscale 4 to those obtained in the general population but differ in scores on the rest of the subscales (M_Scale1_ = 35.73, M_Scale2_ = 32.70, M_Scale3_ = 38.29, M_Scale4_ = 18.73).

## 3 Discussion

The adult attachment style is shaped by a series of cognitive-emotional schemas developed from the bonds that are established throughout life (Feeney and Noller, 1996). Given its influence on intimate relationships, attachment has been extensively studied in research on interpersonal violence. Both research findings and those derived from psychological assessment in the forensic setting play a critical role in the legal decision-making process. Thus, it is important to ensure that the instruments used to measure attachment style are appropriate and provide accurate results and adequate identification of the construct.

The aim of this study was to obtain evidence of validity of the Adult Attachment Scale of Melero and Cantero (2008) in a population of gender-based perpetrators of intimate partner violence. In general, the scale showed adequate psychometric properties to specifically measure the attachment construct in a sample of men convicted of a gender violence crime (IPVAW): good structural validity and internal consistency were confirmed, as well as the formation of profiles that matched attachment styles from the factors of the questionnaire.

Reliability values were adequate, except for the subscale of emotional self-sufficiency and discomfort with intimacy (0.665). The value of the Cronbach's alpha in the original scale was also close to the one found here (0.68). On the other hand, the study of the original scale items showed an optimal discrimination index, except for the items that were eliminated (DI_Item21_ = 0.064; DI_Item11_ = 0.234; DI_Item35_ = 0.234; DI_Item25_ = 0.107). Discrimination indicates whether the item contributes to measuring the same thing that the questionnaire measures (Andrich and Marais, 2019). Low correlations indicate that the item measures something different and it is recommended to eliminate items with correlations close to zero (Abad et al., 2011). The mean values of the items are generally around the theoretical median of the scale, except in factor 3, which indicates that the participants in this study feel confident in intimate relationships and tend to have a high perceived emotional expression. The distribution of responses maintained adequate values (Range_SD_ = 1.02–1.93). In addition, the asymmetry of the items took values between −1.00 and 1.82, and the kurtosis between −1.36 and 3.03. A higher value was observed for item 36 (Sk = 9.33), indicating that there are extremely high values in the distribution, or that the data distribution is skewed to the right in an extreme way.

The combination of the subscales showed the expected attachment style results. The distribution of the four subscales of the questionnaire in the different combinations of profiles performed in ranges and mean values compatible with the original scale in the general population. This data indicates that attachment style is measured with the same dimensions with this population as with the original sample, showing adequate psychometric properties to do so. However, in the 4-subscale model, four of the 40 items did not work correctly in this sample and were eliminated. First, item 21 (“*I have confidence in myself* ”) was eliminated. This item was part of the scale “Low self-esteem, need for approval and fear of rejection” and differed from the rest in that it seemed to measure self-concept from the perspective of the *self* and the others from the external perspective. Self-esteem refers to the evaluation and perception that a person has of himself/herself. It is the subjective assessment of one's own value, competence, and worth as an individual. Self-esteem and fear of rejection are significantly related. It is understood that low self-esteem can make a person feel insecure about his or her worth and fear being rejected or not being accepted by others (Van Tuinen and Ramanaiah, 1979). The results obtained are striking, since other known self-esteem scales, such as Rosenberg's (1965), include appraisals and perceptions about oneself. This difference could be due to the fact that, in the sample studied here, this scale could measure self-esteem as the perception of how we are accepted or rejected by others in our social environment, in line with the postulates of sociometric theory. According to this theory, social interactions and relationships with others play an important role in the formation and maintenance of self-esteem (Leary and Baumeister, 2000). Secondly, something similar happens with items 11 (“*I have trouble asking personal questions*”) and 35 (“*I am a person who prefers solitude to social relationships*”), designed to measure “Emotional expression and confidence in relationships.” It is important to note that these are the only items in the subscale that consider self-perception. Finally, item 25 (“*I prefer stable relationships to sporadic partners*”) was eliminated because it did not adequately capture the factor most linked to avoidance: “Emotional self-sufficiency and discomfort with intimacy.” People with avoidant/dismissing attachment style tend to have difficulty establishing and maintaining close, intimate relationships. They often have a fear of intimacy and tend to avoid emotional dependence in relationships (Edelstein and Shaver, 2004). However, this does not necessarily mean that they prefer sporadic relationships. A study on monogamous relationships (Moors et al., 2015) found that people with an avoidant/dismissing attachment style had more positive attitudes toward consensual non-monogamous relationships and were more willing to engage in them compared to monogamous relationships. However, these individuals were more likely to be involved in monogamous relationships in practice.

Regarding the distribution of attachment style, the results were inconsistent with those obtained in the study of the general population. In our study, 50.57% of the participants showed secure attachment, 4.57% showed avoidant/dismissing attachment, 36.9% showed preoccupied/anxious attachment, and 8.57% showed fearful attachment. In contrast, results from the general Spanish population found a more equal distribution of attachment styles, where 28.54% of subjects were secure, 29.66% avoidant, 26.07% preoccupied, and 15.17% fearful. However, a meta-analysis by Van Ijzendoorn et al. (1999) examined 33 studies on attachment styles based on the Adult Attachment Interview and concluded that the global frequency of the three main attachment styles was as follows: 58% secure, 24% avoidant/dismissing, and 18% preoccupied/anxious. Anxious attachment peculiarities can escalate to hostile masculinity, an issue that increases the likelihood of perpetration in men (Barbaro et al., 2019). The data indicate that the ratio between secure and insecure attachment individuals is equal. However, in the sample studied here, insecure male are mainly anxious. This is not in line with other studies that have evaluated attachment style in perpetrators and have found a greater presence of avoidant attachment among insecure individuals (Lawson and Brossart, 2013). Therefore, it is difficult to establish a predominance of insecure attachment styles in male abusers in the case of our work.

### 3.1 Limitations and future directions

First, the type of sampling used in this investigation could influence the fact that the data collected are not representative of the total population of male perpetrators, the distribution of attachment style was particularly unequal, and does not seem to coincide with the distribution of other studies with the same population. This may indicate a problem of population representativeness or response bias. Second, and related to the previous limitation, the use of self-reporting may introduce response biases, as participants may not be completely honest or accurate in reporting their behaviors and attitudes. In this case, participants are incarcerated and social desirability may be especially present. Third, the reliability of scale 4 fell short of adequate, indicating little consistency in the measurement of avoidance. Fourth, it would be interesting to obtain other validity evidence such as that based on other variables or to perform an invariance analysis.

This work offers an instrument with adequate properties to measure attachment in this population, thus increasing knowledge in this field. In addition, it can guide and specify treatment and prevention components in IPVAW. For future research it will be important to carry out additional studies that provide additional evidence of validity of the original scale in samples of abusers, obtaining convergent, discriminant, predictive or criterion information. These studies would allow us to deepen our understanding of the adult attachment construct and its relevance and peculiarities in this population.

## Data availability statement

The raw data supporting the conclusions of this article will be made available by the authors, without undue reservation.

## Ethics statement

The studies involving humans were approved by Deontological Commission of the Faculty of Psychology of the Complutense University of Madrid. The studies were conducted in accordance with the local legislation and institutional requirements. The participants provided their written informed consent to participate in this study.

## Author contributions

IO-S: Conceptualization, Data curation, Formal analysis, Funding acquisition, Investigation, Methodology, Project administration, Resources, Software, Validation, Visualization, Writing—original draft, Writing—review & editing. AA: Conceptualization, Funding acquisition, Investigation, Project administration, Resources, Supervision, Writing—original draft, Writing—review & editing. PM: Investigation, Resources, Writing—original draft, Writing—review & editing. MD-D: Conceptualization, Supervision, Writing—original draft, Writing—review & editing.
